# Dietary *Bacillus licheniformis* improves the effect of *Astragalus membranaceus* extract on blood glucose by regulating antioxidation activity and intestinal microbiota in *InR*^[E19]^/TM2 *Drosophila melanogaster*

**DOI:** 10.1371/journal.pone.0271177

**Published:** 2022-07-13

**Authors:** Denghui Wang, Yaxin Zhang, Meiling Xu, Xiaoling Sun, Xiulin Cui, Xiuran Wang, Dongbo Liu

**Affiliations:** 1 School of Life Science, Northeast Normal University, Changchun, PR China; 2 School of Food Technology and Biotechnology, Changchun Vocational Institute of Technology, Changchun, PR China; 3 Engineering Research Center of Bioreactor and Pharmaceutical Development, Ministry of Education, College of Life Sciences, Jilin Agricultural University, Changchun, PR China; Inha University, REPUBLIC OF KOREA

## Abstract

**Background:**

The diabetes mellitus prevalence is rapidly increasing in most parts of the world and has become a vital health problem. Probiotic and herbal foods are valuable in the treatment of diabetes.

**Methods and performance:**

In this study, *Bacillus licheniformis* (BL) and *Astragalus membranaceus* extract (AE) were given with food to *InR*^[E19]^/TM2 *Drosophila melanogaster*, and the blood glucose, antioxidation activity and intestinal microbiota were investigated. The obtained results showed that BA (BL and AE combination) supplementation markedly decreased the blood glucose concentration compared with the standard diet control group, accompanied by significantly increased enzymatic activities of catalase (CAT), decreased MDA levels and prolonged lifespan of *InR*^[E19]^/TM2 *D*. *melanogaster*. The treatments with BL, AE and BA also ameliorated intestinal microbiota equilibrium by increasing the population of *Lactobacillus* and significantly decreasing the abundance of *Wolbachia*. In addition, clearly different evolutionary clusters were found among the control, BL, AE and BA-supplemented diets, and the beneficial microbiota, *Lactobacillaceae* and *Acetobacter*, were found to be significantly increased in male flies that were fed BA. These results indicated that dietary supplementation with AE combined with BL not only decreased blood glucose but also extended the lifespan, with CAT increasing, MDA decreasing, and intestinal microbiota improving in *InR*^[E19]^/TM2 *D*. *melanogaster*.

**Conclusion:**

The obtained results showed that dietary supplementation with BL and AE, under the synergistic effect of BL and AE, not only prolonged the lifespan of *InR*^[E19]^/TM2 *D*. *melanogaster*, increased body weight, and improved the body’s antiaging enzyme activity but also effectively improved the types and quantities of beneficial bacteria in the intestinal flora of *InR*^[E19]^/TM2 *D*. *melanogaster* to improve the characteristics of diabetes symptoms. This study provides scientific evidence for a safe and effective dietary therapeutic method for diabetes mellitus.

## Introduction

The prevalence of diabetes mellitus (DM) is rapidly increasing in most parts of the world and has become a vital health problem [1, 2]. Type 2 diabetes mellitus (T2DM) is caused by insulin resistance and the insulin production and secretion decline of β-cells. The current therapies for T2DM only control blood glucose levels; thus, patients always develop tolerance requiring higher medicine doses with more side effects [1, 2]. Therefore, a safe and effective dietary therapeutic method for the prevention and control of T2DM is urgently needed.

*Astragalus membranaceus*, prepared from the roots of *Astragali Radix* known as “Huangqi”, is used in traditional Chinese food and medicine for its beneficial immune-regulatory functions [3, 4]. It has been reported that flavonoids and polysaccharides of *Astragalus* are the primary active components. In particular, the extracts of *A*. *membranaceus* are effective in alleviating clinical symptoms and related complications of diabetes [3–5] and should also work with other traditional Chinese medicines or chemical drugs to achieve complementary advantages, reduce toxicity and increase efficiency [6]. Yang et al. (2021) showed that *A*. *membranaceus* prolongs lifespan by regulating antioxidant ability and insulin/IGF-1 signalling and may be explored as a novel agent for slowing the ageing process [7].

Probiotics can improve gut performance, promote nutrient metabolism, modulate immunity and alter the development and composition of the gut microbiota [8, 9]. Metagenomic analysis of the gut microbiota suggests that the microbiota is altered in patients with type 2 diabetes (T2D) [10–13]. For type 2 diabetes, strategies were developed by selecting promising probiotic microbial strains [13, 14].

*Bacillus licheniformis* is a probiotic that is widely used in enteritis, diarrhoea and intestinal endotoxaemia therapy with no evident adverse reactions [15]. Previous studies have demonstrated that *B*. *licheniformis* supplementation could improve the growth parameters of animals [15, 16]. Since 2007, the European Food Safety Authority (EFSA) has designated 12 *Bacillus* species, including *B*. *licheniformis*, as coming under the qualified presumption of safety status [17]. Recent studies have shown that *B*. *licheniformis* decreases postprandial hyperglycaemia, counteracting the activity of α-glucosidase and preventing the onset of diabetes [18]. However, there is no report about the effects of *B*. *licheniformis* as a supplement in anti-diabetic treatment. Can supplementation with *B*. *licheniformis* improve the extracts of *A*. *membranaceus* for diabetes treatment? If there is a complementary advantage, the obtained results may provide guidance for improving diabetes symptoms.

*Drosophila* and mammals have a high degree of similarity in terms of molecular and biological characteristics [19, 20]. In the last two decades, *Drosophila melanogaster* has been extensively used to elucidate the mechanisms of beneficial host-gut microbiota interactions. As a burgeoning material, *D*. *melanogaster* is frequently used for intestinal microbiota and metabolism research [19–21]. As a model, *D*. *melanogaster* is much easier to maintain in large quantities due to its body size and lifespan [22–24]. Therefore, *D*. *melanogaster* is often used to investigate immune regulation, diet, and probiotic characteristics that are related to ageing [22, 23]. Furthermore, flies can achieve homeostasis via the insulin signalling pathway, *Drosophila* with *InR* defects (insulin receptor mutant) showed significantly lower insulin tolerance [21, 22], and they can serve as a screening model for potential probiotic foods for diabetes management [25, 26].

This study aims to clarify the influence of *B*. *licheniformis* and the extracts of *A*. *membranaceus* or a combination of both on the lifespan, antioxidants, blood glucose and gut microbial community structure of *InR*^[E19]^/TM2 *D*. *melanogaster*, which may help us to evaluate the interaction between the probiotics and the extracts of *A*. *membranaceus* in insulin deficiency. This work will provide the possible antidiabetic mechanisms of *B*. *licheniformis* and the extract of *A*. *membranaceus* in *InR*^[E19]^/TM2 *D*. *melanogaster*.

## Materials and methods

### Drosophila husbandry

*InR*^[E19]^/TM2 *D*. *melanogaster* (#9646) was purchased from Bloomington *Drosophila* Stock Center (Indiana University, Bloomington, IN, USA). *InR*^[E19]^/TM2 is an inducible mutation model induced by ethyl methanesulfonate exposure. In this study, male flies were selected after anaesthetization with CO_2_ and emerged within 8 h without mating. The yeast-sucrose-cornmeal diet included 9 g of soybean meal, 66.825 g of corn flour, 30 g of sucrose, 0.5 g of sodium benzoate, 25 g of yeast, 6.5 g of agar, and 6.8 mL of propionic acid per litre. *D*. *melanogaster* was grown at 25°C and 65% humidity with a 12 h:12 h light-dark cycle.

In this study, the following four groups were used: standard diet group (control), *A*. *membranaceus* extract (AE) group, *B*. *licheniformis* (BL, 1 × 10^8^ CFU mL^-1^) group, and *B*. *licheniformis* and *A*. *membranaceus* extract (BA) group.

### Microorganism and culture conditions

The *B*. *licheniformis* strain was isolated with LB medium from the probiotic product Zhengchangsheng (Northeast Pharmaceutical Group Shenyang No.1 Pharmaceutical Co., Ltd, Shenyang, China), which is an innovative probiotic drug containing *B*. *licheniformis* bacteria for acute and chronic enteritis and diarrhoea therapy. It is a microbial pharmaceutics approved by the China Food and Drug Administration (Permission Number: S10950019). The bacteria were cultured at 37°C and 180 rpm for 18 h. The bacteria were added to the diets (40°C) at 1×10^8^ CFU mL^-1^.

### Chemicals

The final concentration of *A*. *membranaceus* extract was 2%. *A*. *membranaceus* extract: 100 g of *A*. *membranaceus* was added to 500 mL of distilled water and soaked in warm water for 3 h with constant stirring to ensure adequate soaking. Then, 500 mL of water was added, boiled at 100°C for 1 h, and filtered with double gauze for retention. Then, 1 L of water was added to the remaining residue, boiled for 1 h, filtered with double gauze for retention, combined twice to retain the liquid, and concentrated to a 10% concentration of *A*. *membranaceus* decoction (1 L).

### Lifespan assay

A total of 480 male virgin flies that emerged and did not mate within 8 h were selected and divided into 4 groups under CO_2_ anaesthesia, with 6 bottles in each group and 20 in each bottle. They were put into the corresponding test tube, and the test tube mouth was sealed with a cotton plug made of sterilized absorbent cotton. At the same time, all fruit flies were cultured in a light dark cycle for 12 h under constant temperature (25°C) and relative humidity (60%). The culture medium was changed once every 5 days, and the survival number of *Drosophila* was observed and recorded at 8:30 every day until the last *Drosophila* died. *Drosophila* melanogaster that died accidentally due to excessive anaesthesia or other human factors was not included. According to the counting results, the lifespan of fruit flies in each group was analysed.

### Body weight

The body weight of *Drosophila melanogaster* was recorded on the 10^th^, 20^th^ and 30^th^ day. Each group of fruit flies was anaesthetized with CO_2_ gas, and then the weight of each group of samples was measured with an ultrasensitive electronic analytical balance (Mettler Toledo AL204). The average weight of fruit flies in each group was calculated. Then, those flies were stored at -80°C for later enzyme and intestinal microbiota assays.

### Analysis of CAT activity, MDA and blood glucose levels

The preserved *Drosophila melanogaster* were washed with 70% ethanol and sterile phosphate buffered saline 3 times, and then 9 times normal saline was added according to the ratio of weight (g): volume (mL) = 1:9. The tissue was ground, the homogenate was prepared in an ice water bath and centrifuged at 3000 rpm for 10 minutes, and the supernatant was collected to obtain 10% homogenate supernatant. Antioxidant activity changes in homogenate supernatant were evaluated by catalase (CAT) with a CAT assay kit (B518131, Nanjing Jiancheng Bioengineering Institute, China). Because the absorbance of hydrogen peroxide reached the maximum value at 240 nm, the CAT activity was expressed as the decomposition of H_2_O_2_ as measured by the decrease in absorbance at 240 nm for 3 min. CAT activity was expressed on a protein basis, and the protein content of the enzyme extract was determined using bovine serum albumin (BSA) as a standard according to the method described by Bradford (1976) [27]. The malondialdehyde (MDA) level of the homogenate supernatant was quantified using the thiobarbituric acid (TBA) method (B518191, Nanjing Jiancheng Bioengineering Institute, China). Briefly, homogenate supernatant was reacted with a solution mixed with 5% trichloroacetic acid (TCA) and 0.5% TBA in 0.2 mol L^-1^ HCl. The sample was incubated in a 95°C water bath for 40 min and then cooled to room temperature. After centrifugation at 3500 rpm for 10 min, the supernatant absorbance was determined at 532 nm, and 1,1,3,3-tetramethoxypropane was used as a standard. Blood glucose levels of *InR*^[E19]^/TM2 *D*. *melanogaster* were measured according to the method described by Tennessen et al. (2014) [28].

### 16S rDNA high-throughput sequencing

Flies from days 10, 20 and 30 were rinsed in 50% bleach, followed by 70% ethanol, and then washed with sterile PBS before dissection. Samples were dissected from 20 flies in phosphate buffered saline. The intestines were collected after removing the Malpighian tubules and trachea under a stereomicroscope. Genomic DNA was extracted using a QIAGEN DNeasy® Blood and Tissue Kit (Qiagen, 69504). The genomic DNA was sent to Novogene (Beijing, China) for sequencing by Illumina MiSeq and sequence library construction. The 16S rRNA amplicon sequencing was conducted using the Illumina MiSeq (300-bp paired-end reads) platform. The extracted DNA (20–30 ng μL^-1^) was used as the template to amplify the V3-V4 region of the 16S rRNA gene in triplicate using the primer set 515F (5′-GTGCCAGCMGCCGCGGTAA-3′) and 806R (5’-GGACTACHVGGGTWTCTAAT-3′). The polymerase chain reaction (PCR) was performed in a 30-μL reaction system that contained 15 μL of 2 × PCR buffer, 1 μL of 0.2 μmol L^-1^ of each primer, 0.5 μL of Taq DNA enzyme, 1 μL of template DNA within, and 2 μL of ddH_2_O. The thermal cycle conditions consisted of initial denaturation at 98°C for 1 min, followed by 30 cycles of 10 s at 98°C, 30 s at 50°C, and 30 s at 72°C, then finally ending with a final extension of 5 min at 72°C. PCR amplification was performed using an ABI GeneAmp® 9700 PCR instrument. The 16S rRNA reference database and sequence clustering were aligned by VSEARCH (1.9.6) and Silva 132. Operational taxonomic unit (OTU) clustering was performed for unique sequences with 97% similarity. During the clustering process, chimeric sequences were removed to obtain the representative sequences of OTUs. Random sampling was used to calculate the Shannon and Chao1 α diversity index according to the OTU analysis. Weighted UniFrac analysis was applied to compare differences in microbial communities. Beta diversity was visualized by principal coordinate analysis (PCoA) and nonmetric multidimensional scaling (NMDS). In addition, redundancy analysis (RDA) was performed by the Hellinger method. PICRUSt (phylogenetic investigation of communities by reconstruction of unobserved states) and Kyoto Encyclopedia of Genes and Genomes (KEGG) orthologues were used to evaluate the bacterial functional genes. All data have been uploaded to the NCBI database (PRJNA838651).

### Statistical analysis

All data (lifespan, body weight, CAT activity, MDA and blood-glucose content) were collected by GraphPad Prism 8 software (GraphPad Software, La Jolla, CA, USA) and analysed by IBM SPSS Statistics (Version 25, IBM SPSS, Chicago, IL, USA). PICRUSt analysis was performed on the Galaxy platform (http://huttenhower.sph.harvard.edu/galaxy/). The KEGG orthologues were analysed by Metagenomic Profiles (STAMP) software (Dalhousie University, Halifax, NS, Canada). Two-tailed Student’s t-tests were used to compare samples, and asterisks indicate statistical significance (*p < 0.05, **p < 0.01, ***p < 0.001 and ****p < 0.0001).

## Results

### Lifespan analysis

**Fig 1:**
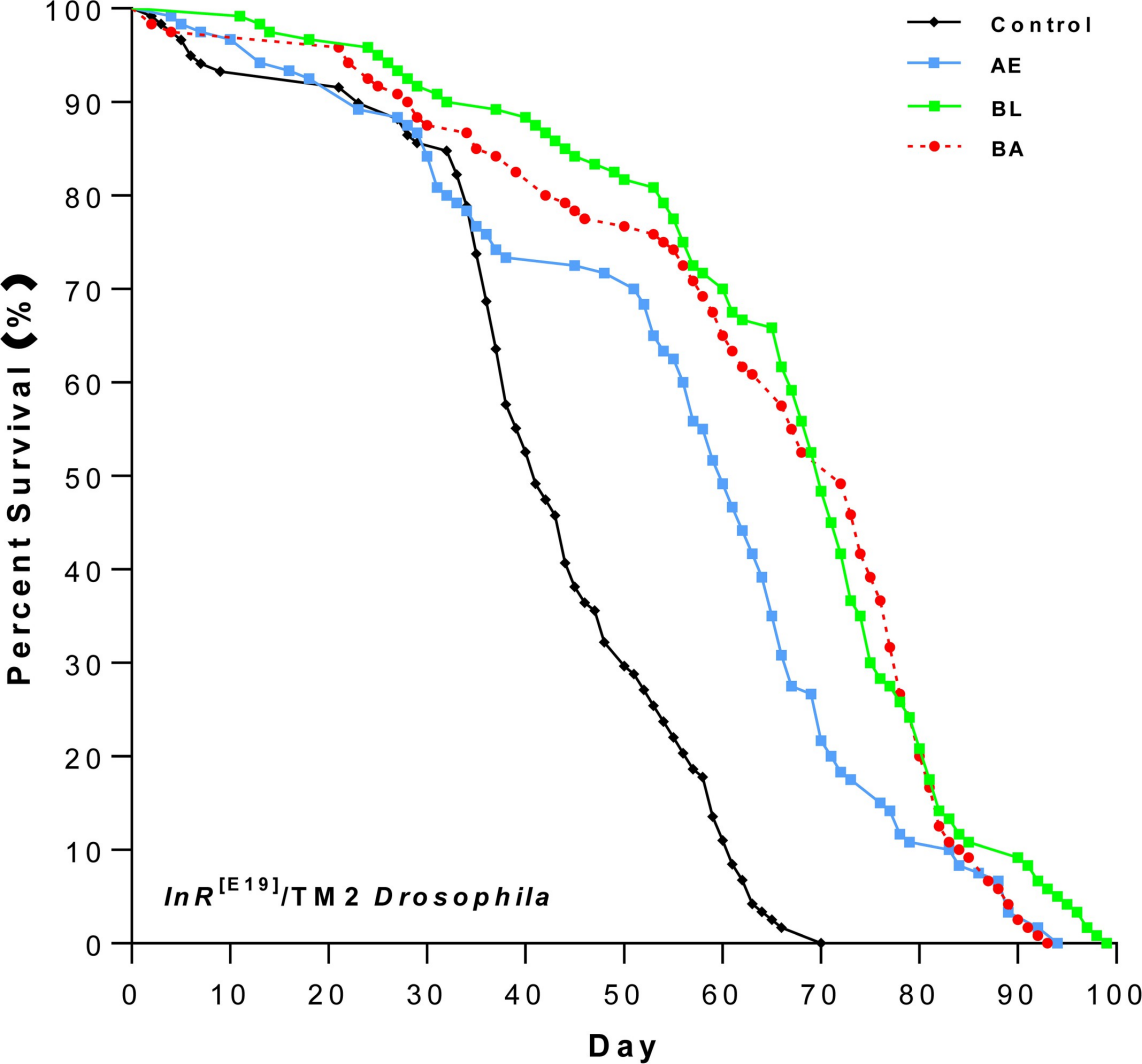
Lifespan curves of *InR*^[E19]^/TM2 *D*. *melanogaster* in four groups. AE: *Astragalus membranaceus* extract, BL: *Bacillus licheniformis* and BA: *Bacillus licheniformis* and *Astragalus membranaceus* extract.

The lifespan of male *Drosophila* (*InR*^[E19]^/TM2) that were fed BL, AE, and BA showed significant differences compared to that of the control (Fig 1). The Kaplan–Meier test of survival curves demonstrated increased longevity for BL (p < 0.0001), AE (p < 0.0001) and BA (p < 0.0001, S1 Table). Compared to the control group, *InR*^[E19]^/TM2 *D*. *melanogaster* in the AE, BL and BA groups increased their mean lifespan by 32.55%, 45.31% and 34.29%, median lifespan by 56.81%, 69.10% and 44.43% and maximum lifespan by 51.19%, 74.25% and 32.86%, respectively (S1 Table). The longevity-promoting activities of BA and BL were higher than those of AE (Fig 1).

### Body weight

**Fig 2:**
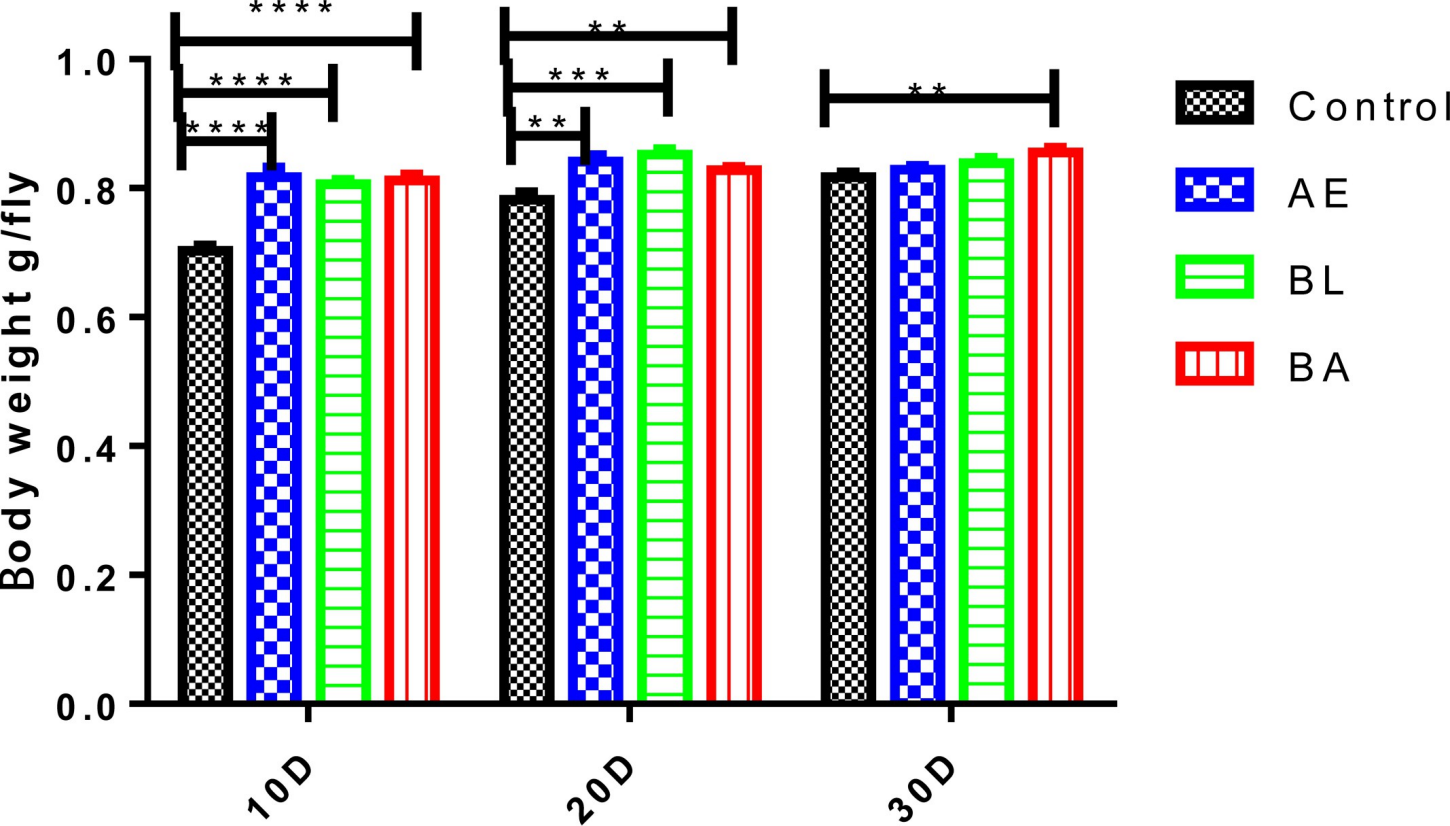
Body weight of *InR*^[E19]/^TM2 *D*. *melanogaster* on days 10 (10D), 20 (20D), and 30 (30D) in the four groups. AE: *Astragalus membranaceus* extract, BL: *Bacillus licheniformis* and BA: *Bacillus licheniformis* and *Astragalus membranaceus* extract. 10D, 20D and 30D represent *InR*^[E19]^/TM2 *D*. *melanogaster* growth at 10, 20 and 30 days in each group, respectively.

On the 10^th^ and 20^th^ days, the body weight of *InR*^[E19]^/TM2 *D*. *melanogaster* in the AE, BL and BA groups was significantly higher than that in the control group (all p < 0.05), but on the 30^th^ day, the body weight of *InR*^[E19]^/TM2 *D*. *melanogaster* was significantly increased in the BA group compared to that in the control group (p < 0.05, Fig 2).

### CAT activity, MDA and blood glucose content

**Fig 3:**
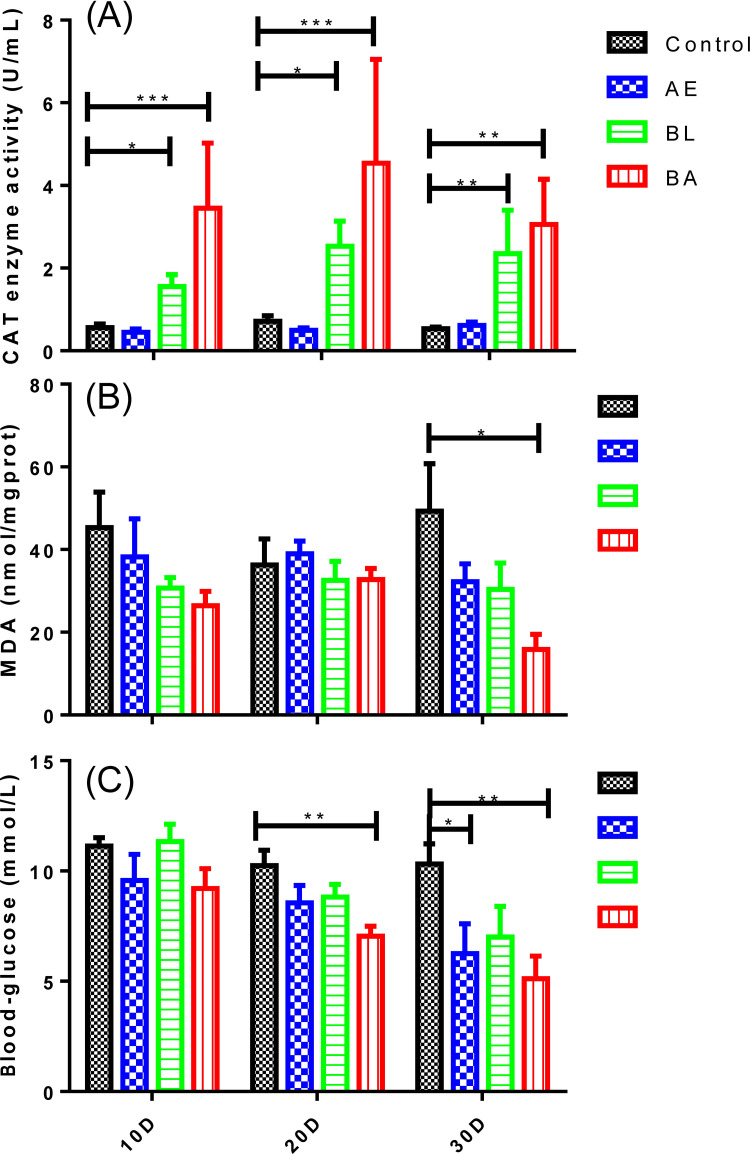
CAT activity (A), MDA content (B) and blood glucose content (C) of *InR*^[E19]^/TM2 *D*. *melanogaster* on days 10 (10D), 20 (20D), and 30 (30D) in the four groups. AE: *Astragalus membranaceus* extract, BL: *Bacillus licheniformis* and BA: *Bacillus licheniformis* and *Astragalus membranaceus* extract. *p < 0.05, **p < 0.01, ***p < 0.001 indicates significance between the control and experimental groups.

From the 10^th^ to 30^th^ day, the CAT enzyme activity of *InR*^[E19]^/TM2 *D*. *melanogaster* in the BA and BL groups was significantly higher than that in the control (Fig 3A). On day 30, the MDA content of *InR*^[E19]^/TM2 *D*. *melanogaster* in the BA group was significantly decreased compared to that in the control group (Fig 3B). From the 20^th^ to 30^th^ day, the blood glucose concentration of *InR*^[E19]^/TM2 *D*. *melanogaster* in the BA group was significantly decreased compared to that in the control, and that in the AE group was significantly decreased compared to that in the control at day 30 of growth in *InR*^[E19]^/TM2 *D*. *melanogaster* (Fig 3C).

### Gut microbiota

**Fig 4:**
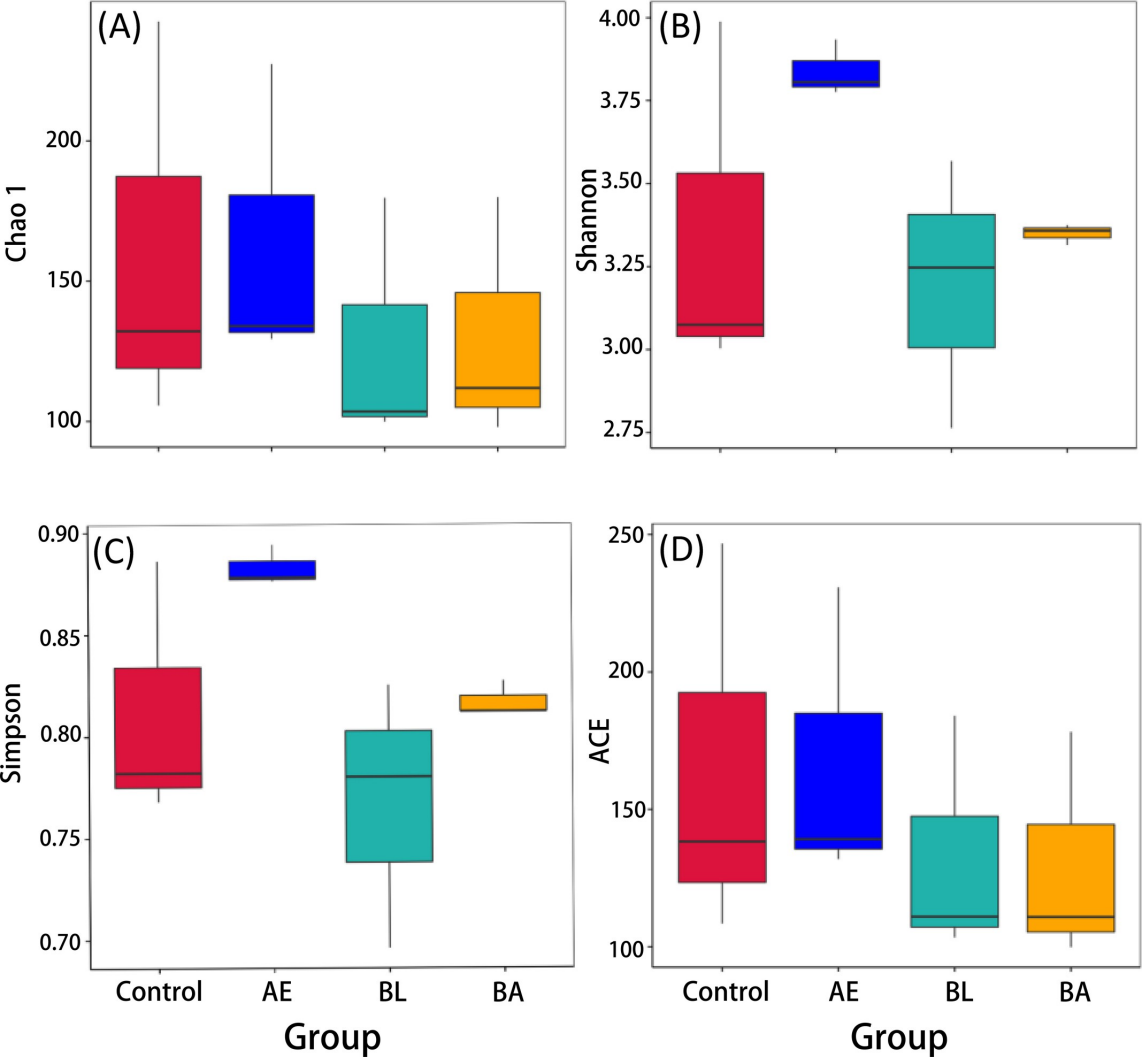
Gut microbiota diversity indices of *InR*^[E19]^/TM2 *D*. *melanogaster* in the four groups. AE: *Astragalus membranaceus* extract, BL: *Bacillus licheniformis* and BA: *Bacillus licheniformis* and *Astragalus membranaceus* extract.

**Fig 5:**
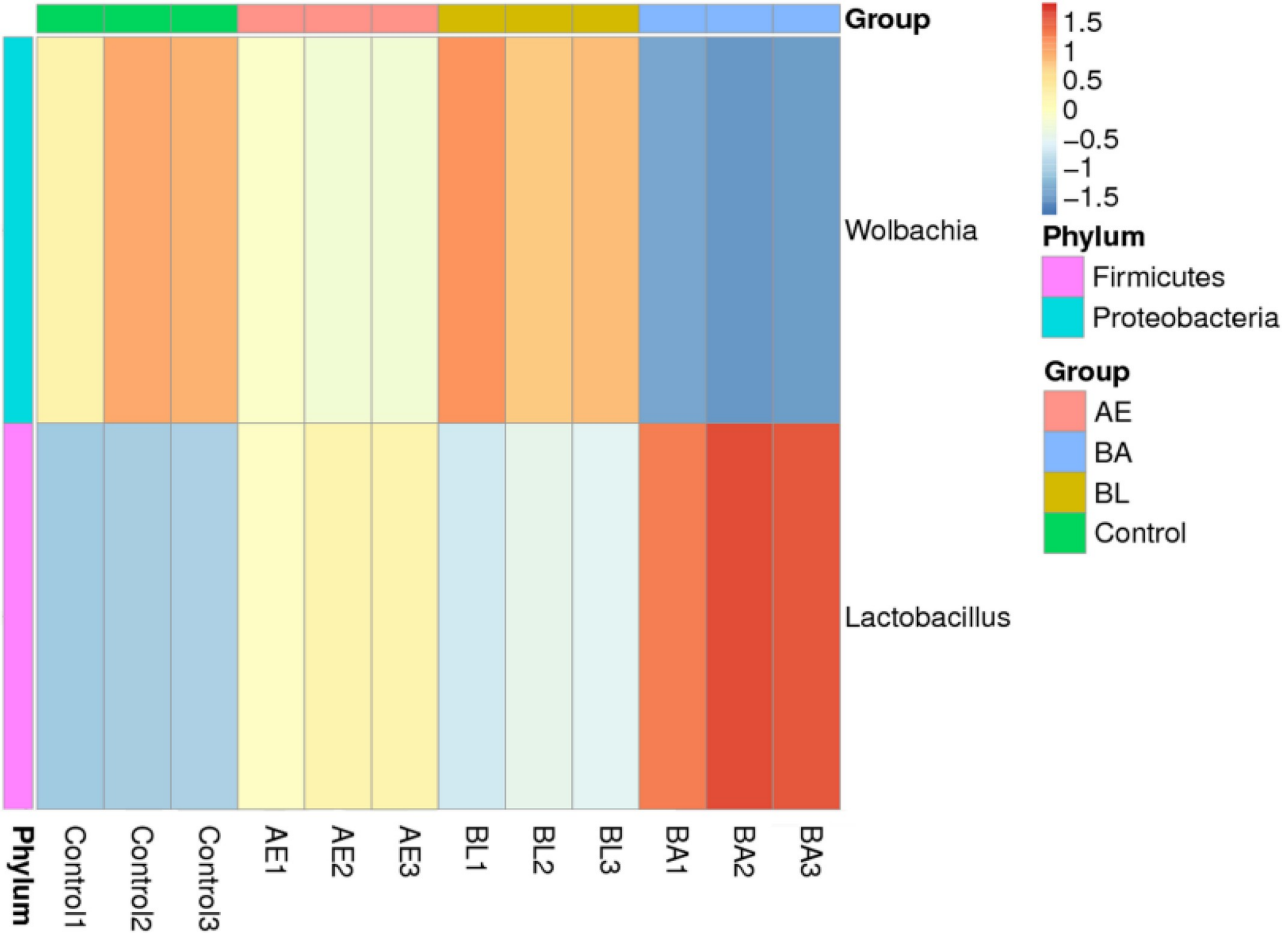
Relative abundance of *Acetobacter*, *Wolbachia*, and *Lactobacillus* in the four groups. AE: *Astragalus membranaceus* extract, AE1, AE2 and AE3 represent the three technical replicates of the AE group, BL: *Bacillus licheniformis*, BL1, BL2 and BL3 represent the three technical replicates of the BL group, BA: *Bacillus licheniformis* and *Astragalus membranaceus* extract, BA1, BA2 and BA3 represent the three technical replicates of the BA group.

In total, 4,473,148 sequences were obtained, and 2,236,574 clean sequences were detected for subsequent analysis. The alpha diversities of gut microbiota in *InR*^[E19]^/TM2 *D*. *melanogaster* in the control, BL, AE and BA groups are shown in Fig 4. Compared to the control, the species evenness and richness of the gut microbiota in flies were not significantly enhanced in the BL, AE and BA groups, according to Shannon, Chao1, ACE and Simpson effective counts. At day 30, BL, AE and BA markedly increased the abundance of *Lactobacillus* (p = 0.006; p = 0.003; p = 0.004) (Fig 5). Furthermore, *Wolbachia* was found to be significantly reduced in the AE and BA groups at day 30 (p = 0.009; p = 0.003) (Fig 5).

**Fig 6:**
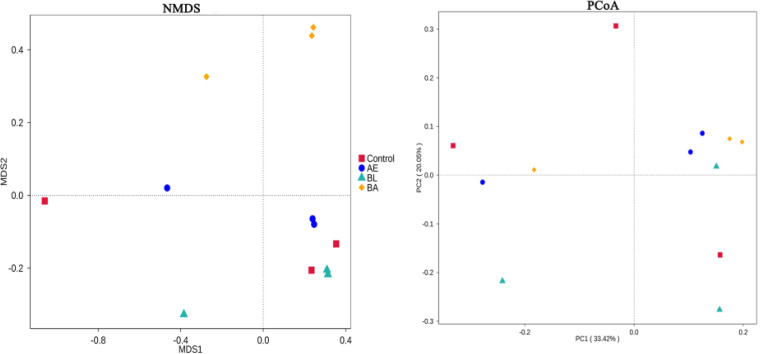
NMDS analysis and PCoA showing the shifts in the bacterial communities driven by BL, AE and BA among all samples. AE: *Astragalus membranaceus* extract, BL: *Bacillus licheniformis* and BA: *Bacillus micheniformis* and *Astragalus membranaceus* extract.

**Fig 7:**
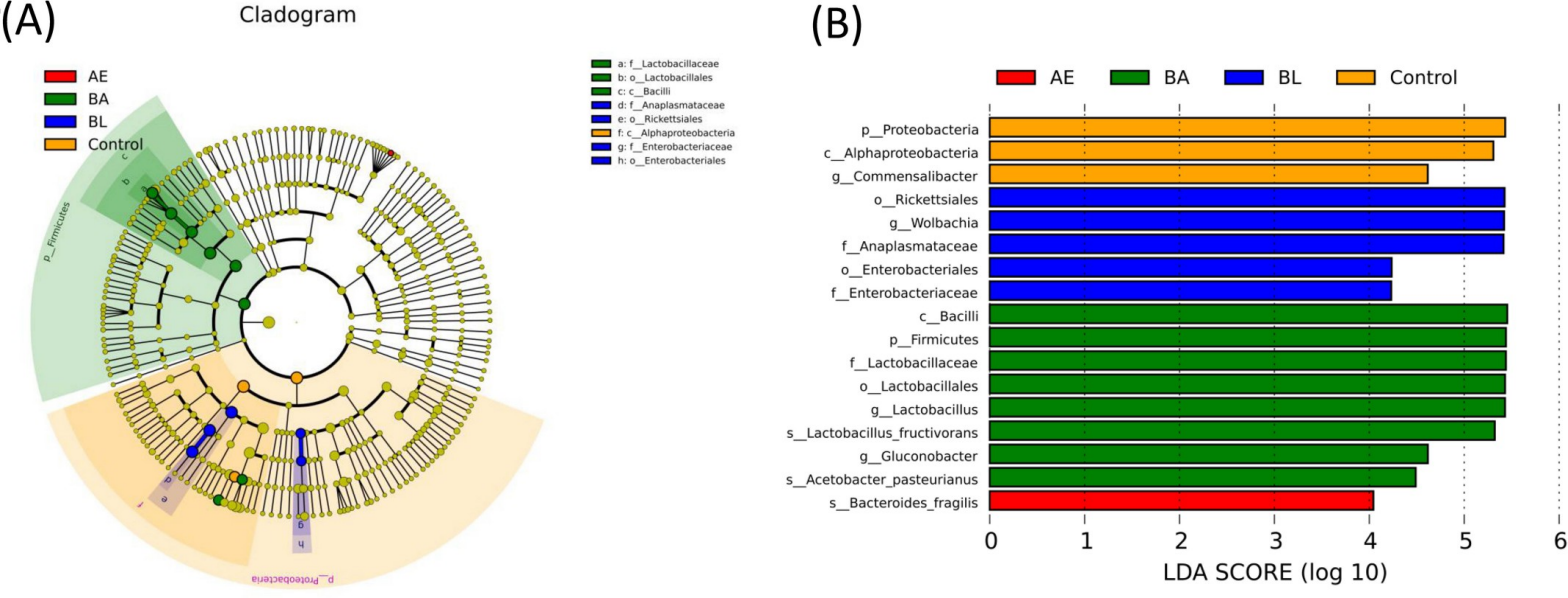
Cladogram (A) and linear discriminant analysis (LDA, B) scores of the *InR*^[E19]^/TM2 *D*. *melanogaster* on the AE, BL, BA-supplemented diet and the standard (Control) diet. AE: *Astragalus membranaceus* extract, BL: *Bacillus licheniformis* and BA: *Bacillus*. *licheniformis* and *Astragalus membranaceus* extract.

The NMDS and PCoA analyses revealed that the microbial communities of the control, BL, AE and BA groups were clustered separately from each other (ANOSIM R = 0.98, p = 0.001) at day 30 (Fig 6). LEfSe analysis was used to identify overrepresented OTUs and compare their relative abundances in different groups. As shown in Fig 7, evolutionary clusters were clearly different from those in the control, BL, AE and BA-supplemented diets. Among the beneficial microbiota, *Lactobacillaceae* and *Acetobacter* were found to remarkably increase in male flies that were fed BA, and *Enterobacteriaceae* was enriched in the BL group; *Alphaproteobacteria* was relatively higher in the control diet group.

## Discussion

The study investigated the effects of BL supplied with AE as diet supplements on blood glucose in *InR* ^[E19]^/TM2 *D*. *melanogaster*. The obtained results revealed that the blood glucose concentration significantly decreased in the BA groups, accompanied by an increase in the lifespan of *InR*^[E19]^/TM2 *D*. *melanogaster* on day 30. The mechanism of AE- and BL-mediated blood glucose levels and lifespan extension of *InR*[^E19]^/TM2 *D*. *melanogaster* were explored by measuring changes in antioxidant capacity and the gut microbiota.

*A*. *membranaceus* has been reported to have a variety of functions, such as enhancing immunity, resisting ageing, lowering blood glucose and preventing lipid peroxidation [29–31]. AEs have been proven to be a safe healthy food in China, and we can use AEs to prevent diabetic symptoms [3–5, 17]. Our results showed that the BA group significantly improved blood glucose parameters; the results were even better than those in the control positive group in the *InR*^[E19]^/TM2 *D*. *melanogaster* model. At day 30, the blood glucose concentration of *InR*^[E19]^/TM2 *D*. *melanogaster* in the AE group was also decreased compared with that in the control. When AE was supplied with BL, the blood glucose concentration of *InR*^[E19]^/TM2 *D*. *melanogaster* in the BA group was significantly decreased compared with that in the control group from day 20. This result shows that the positive effect of AE combined with BL improved diabetic symptoms better than BL and AE, while further investigation is needed to explore the mechanisms of AE coordinated with BL.

The effect of *A*. *membranaceus* crude extract on lifespan was assessed. As shown in Fig 1, AE can significantly prolong the lifespan of fruit flies, which is consistent with previously reported results [4, 29]. Intriguingly, the effects of the BL and BA targets were stronger than those of the AE and control targets. This result suggests that the AE in fruit flies is correlated with the prolonging lifespan capacity, and BL can also improve the capacity, which provides another evidence to explain the beneficial effects of *B*. *licheniformis* on *A*. *membranaceus* towards *InR*^[E19]^/TM2 *D*. *melanogaster*.

The underlying mechanism by which AE and BL prolong lifespan may be related to free radical scavenging. Generally, organism deterioration is caused by free radical species, while antioxidants can overcome this issue [32, 33]. The radical scavenging activity of BL and AE is always related to antioxidant enzymes: CAT detoxifies hydrogen peroxide and converts lipid hydroperoxides to nontoxic substances. The antioxidant enzyme activities were assayed to determine the antioxidant properties of BL and AE. BL treatment significantly enhanced CAT activity and decreased the MDA level, which is a product of lipid peroxidation caused by free radicals in vivo. In this study, we found a significant increase in CAT activity in BL and BA fed *InR*^[E19]^/TM2 *D*. *melanogaster*, suggesting that consuming BL or BA has the potential to clean up free radical molecules in *InR*^[E19]^/TM2 *D*. *melanogaster* [34]. MDA, the product of lipid peroxidation, causes structural and functional alterations associated with ageing organs [35]. BL treatment significantly increased the antioxidant enzyme CAT activity and decreased the MDA level. When AE was combined with BL, it improved CAT activity and further decreased MDA (Fig 3). We suggest that this may be an important reason contributing to the BL- and AE-mediated blood glucose decrease and prolongation of lifespan in *Drosophila*. Further studies will be conducted to evaluate potential molecular markers of lifespan-increasing properties in other animal models.

The comparison of lifespan has been accepted as an important protocol for examining the physiological effects of substances on animal models [19–21]. In this study, dietary supplementation with BL or AE was proven to prolong the lifespan of *InR*^[E19]^/TM2 *D*. *melanogaster* (S1 Table, Fig 1). As beneficial living microorganisms, probiotics always survive in the gastrointestinal tract and play important roles in balancing the intestinal flora, involving barrier defence and mucosal immunity [8, 9]. *B*. *licheniformis* has been widely used as a supplement for animal feed, human food and medical treatment [15, 36, 37]. For example, dietary supplementation with *B*. *licheniformis* improved animal performance and eased necrotic enteritis induced by *Clostridium perfringens* [15, 16]. Recently, *B*. *licheniformis* Dahb1 was reported to increase the growth and antioxidant activity of catfish *Pangasius hypophthalmus* [38]. Midhun et al. reported that the *B*. *licheniformis* strain HGA8B possessed in vitro probiotic activity [39, 40]. In addition, the *B*. *licheniformis* preparation could relieve radiation therapy-related gastrointestinal symptoms and inflammatory reactions in central nervous system tumour (CNST) patients during radiotherapy [41].

Metagenome analyses demonstrated that the intestinal microbiota was modified in type 2 diabetes (T2D), and significant differences were observed between the patient and control groups. Impaired glucose metabolism is related to changes in the bacterial community, and several attempts have been made to target the gut microbiota [10–13]. Many experiments have proven that gut microbiota diversity can be modulated [42, 43]. In this study, the results of 16S amplicon analysis implied that BL, AE and BA might affect the diversity of commensal microbes (Fig 4). Compared to the control, the species evenness and richness of the gut microbiota in flies were not significantly enhanced in the BL, AE and BA groups, according to Shannon, Chao1, ACE and Simpson effective counts. However, the distinct clusters of microbial communities among BL, AE, BA and the control demonstrated an alteration in the beta diversity of intestinal flora, and this may be the factor that affects the lifespan of *InR*^[E19]^/TM2 *D*. *melanogaster*.

The bacterial genus *Lactobacillus* is a vital bacterium in the intestinal flora of *Drosophila* that promotes the growth and development of *Drosophila* [44, 45]. *Lactobacillus* has been reported to regulate the insulin/insulin-like growth factor-like signalling pathway and act as a *Drosophila* nutrient sensing system to modulate host homeostasis, growth rate and individual size [44, 45]. In this study, we found that all treatments markedly increased the abundance of *Lactobacillus* at day 30 (Fig 5). It has been reported that *Lactobacillus reuteri* strains can regulate T2D-related factors and improve insulin resistance in a rat model [46]. Balakumar et al. (2018) demonstrated that the *Lactobacillus* strain combination could improve insulin sensitivity in mice [47]. The results of this study revealed that the abundance of the *Lactobacillus* genus was higher in the gut content of the *B*. *licheniformi*s-treated group, and the abundance of the *Lactobacillus* genus was positively correlated with that of *B*. *licheniformis*, which implied that probiotic supplementation promotes the beneficial bacterial population and, thus, maintains a healthier intestinal system, thereby improving the growth lifespan and body weight parameters. Additionally, *Lactobacillaceae* and *Acetobacter* were found to be significantly increased in flies fed BA. As the dominant microbial genus in *Drosophila melanogaster*, *Acetobacter* species are commonly acetic acid-producing bacteria, which may help regulate host glucose levels, accelerate host development, and increase body size and growth rate in *D*. *melanogaster* [44, 48, 49]. It has been described that *Acetobacter* spp. also influenced the nutrient-sensing insulin/insulin-like growth factor signalling pathways through acetic acid [44]. In the BA group, the increasing abundance of the *Acetobacter* genus suggested the influence of the dietary supplementation of BL and AE combination on improving lifespan of *InR*^[E19]^/TM2 *D*. *melanogaster*.

The results presented in this work indicated that *Wolbachia* was significantly reduced in the AE and BA groups at day 30 (p = 0.009; p = 0.003) (Fig 5). *Wolbachia* bacteria are intracellular bacteria present in the insect and parasitic nematode microbiota. *Wolbachia* bacteria have been shown to influence the reproduction, development and lifespan of insect biology [50, 51]. Although *Wolbachia* has been reported to affect the longevity of hosts, some inconsistency remains [52, 53]. For example, *Wolbachia* was reported to substantially increase the lifespan and competitiveness of infected females under laboratory conditions [52]; nevertheless, some *Wolbachia* strains, such as MelPop, have been shown to shorten the lifespan of *Drosophila* [53]. Interestingly, our results showed that BL, AE and BA significantly reduced the proportion of *Wolbachia* in intestinal flora, and more research is needed to confirm the relationship between decreased *Wolbachia* and lifespan extension of male *InR*^[E19]^/TM2 *D*. *melanogaster*.

## Conclusion

In this study, *Bacillus licheniformis* and *Astragalus membranaceus* extracts were given with food to *InR*^[E19]^/TM2 *D*. *melanogaster*, and the blood glucose, antioxidation activity and intestinal microbiota were investigated. Our results showed that simultaneous feeding of *InR*^[E19]^/TM2 *D*. *melanogaster B*. *licheniformis* and *A*. *membranaceus* extracts had a positive effect on lifespan, body weight, and antioxidant capacity, had a negative effect on MDA levels and blood glucose, and significantly altered the gut microbial community structure, suggesting that *B*. *licheniformis* and *A*. *membranaceus* extracts have potential as an age retardant and therapeutic treatment for diabetes.

## Supporting information

S1 Table Total number of flies, percentage changes of mean and median lifespan, and the log-rank tests. AE: *Astragalus membranaceus* extract, BL: *Bacillus licheniformis* and BA: *Bacillus*. *licheniformis* and *Astragalus membranaceus* extract.
(DOCX)

S1 Data (XLSX)
