# Supplementary material for: Dietary Bacillus licheniformis improves the effect of Astragalus membranaceus extract on blood glucose by regulating antioxidation activity and intestinal microbiota in InR[E19]/TM2 Drosophila melanogaster
Source: PLoS One. 2022 Jul 13;17(7):e0271177. doi: 10.1371/journal.pone.0271177 (PMC9278782; doi:10.1371/journal.pone.0271177)
Supplement: S1 Table — AE: Astragalus membranaceus extract, BL: Bacillus licheniformis and BA: Bacillus. licheniformis and Astragalus membranaceus extract. (DOCX) [file pone.0271177.s001.docx]

**Supporting information**

**Table S1** Total number of flies, percentage changes of mean and median lifespan, and the log-rank tests. AE: *Astragalus* *membranaceus* extract, BL: *Bacillus licheniformis* and BA: *Bacillus. licheniformis* and *Astragalus membranaceus* extract.

|  | **Total n. of flies** | **Mean**  **(% change)** | **Median**  **(% change)** | **maximum**  **(% change)** | **Log-rank**  **(vsstandard diet)** |
| --- | --- | --- | --- | --- | --- |
| **CK** | 118 | 42.12 | 41.75 | 70 | - |
| **AE** | 120 | 55.83 (32.55%) | 60.67(45.31%) | 94(34.29%) | *P*< 0.0001 |
| **BL** | 120 | 66.05 (56.81%) | 70.60(69.10%) | 99(44.43%) | *P*< 0.0001 |
| **BA** | 120 | 63.68 (51.19%) | 72.75 (74.25%) | 93(32.86%) | *P*< 0.0001 |
